# 

**Published:** 1889-12

**Authors:** 


### Bristol Medico-Chirurgical Journal.

DECEMBER, 1889.

### CONTENTS.

ORIGINAL PAPERS: *Page*

Some Surgical Aspects of Carcinoma. By NELSON C. DOBSON, F.R.C.S. Eng. ... ... ... ... ... ... ... ... ... ... ... ... 225

Inebriety. By ALFRED J. H. CRESPI ... ... ... ... ... ... ... ... 244

CLINICAL RECORDS:

A Case of Myxœdema. Congenital Heart Disease. Disseminated Sclerosis. By J. MICHELL CLARKE, M.A., M.B., M.R.C.P. ... 258

REVIEWS OF BOOKS ... ... ... ... ... ... ... ... ... ... ... ... ... ... 267

NOTES ON PREPARATIONS FOR THE SICK ... ... ... ... ... ... ... ... 276

PERISCOPE ... ... ... ... ... ... ... ... ... ... ... ... ... ... ... 280

SCRAPS ... ... ... ... ... ... ... ... ... ... ... ... ... ... ... ... 287

